# Comparison of stroke care parameters in acute ischemic stroke patients with and without concurrent Covid-19. A Nationwide analysis

**DOI:** 10.1186/s42466-020-00095-9

**Published:** 2020-11-19

**Authors:** Daniel Richter, Christos Krogias, Jens Eyding, Dirk Bartig, Armin Grau, Ralph Weber

**Affiliations:** 1 Department of Neurology, St. Josef-Hospital Bochum, Ruhr University Bochum, Gudrunstr. 56, 44791 Bochum, Germany; 2 Medical Faculty, Ruhr University of Bochum, Bochum, Germany; 3 Department of Neurology, Gemeinschaftskrankenhaus Herdecke, Herdecke, Germany; 4 Department of Neurology, Klinikum der Stadt Ludwigshafen, Ludwigshafen, Germany; 5 Department of Neurology, Alfried Krupp Krankenhaus Essen, Essen, Germany

**Keywords:** Covid-19, Stroke, Thrombolysis, Thrombectomy

## Abstract

**Background:**

Comparing health care parameters of acute ischemic stroke (AIS) patients with and without concurrent coronavirus disease 2019 (Covid-19, SARS-CoV-2 infection), may be helpful in terms of optimizing clinical and public health care during pandemic.

**Methods:**

We evaluated a nationwide administrative database of all hospitalized patients with main diagnosis of acute ischemic stroke with/without diagnosis of Covid-19 who were hospitalized during the time period from January 16th to May 15th, 2020. Data from a total of 1463 hospitals in Germany were included. We compared case numbers, treatment characteristics (intravenous thrombolysis, IVT; mechanical thrombectomy, MT; treated on an intensive care unit, stroke unit or regular ward) and in-hospital mortality of AIS with and without concurrent diagnosis of Covid-19.

**Results:**

From a total of 30,864 hospitalized Covid-19 patients during the evaluation period in Germany, we identified a subgroup of 213 patients with primary diagnosis of AIS. Compared to the 68,700 AIS patients without Covid-19, this subgroup showed a similar rate of IVT (16.4% vs. 16.5%, *p* = 0.985) but a significantly lower rate of MT (3.8% vs. 7.9%, *p* = 0.017). In-hospital mortality rate was significantly higher in patients with AIS and concurrent Covid-19 compared to non-infected AIS patients (22.5% vs. 7.8%, *p* < 0.001).

**Conclusion:**

These nationwide data point out differences in mortality and medical treatment regime between AIS patients with and without concurrent Covid-19. Since the pandemic is still ongoing, these data draw attention to AIS as a less frequent but often fatal comorbidity in Covid-19 patients.

**To the editor**

It is assumed that patients with Coronavirus disease 2019 (Covid-19, SARS-CoV-2 infection) and acute ischemic stroke (AIS) are treated and monitored differently, although these patients may have a higher risk of stroke compared to patients with other viral infections such as influenza [1]. To evaluate parameters of acute stroke care for Covid-19 patients with AIS, we analyzed nationwide treatment rates for Germany and in-hospital mortality in these patients for the time period January 16th to May 15th, 2020. Case numbers and treatment characteristics of all hospital admissions of AIS (ICD I63) with and without a concurrent diagnosis of Covid-19 (ICD U07.1) were analyzed using the high-quality and validated administrative diagnosis related group database [2] which is relevant for reimbursement of inpatient treatment cost. Statistical differences in categorical or continuous variables between patients were calculated using chi-squared test (χ2) or t-test, respectively.

A total of 30,864 patients with Covid-19 were hospitalized in Germany between January 16th and May 15th, 2020. We identified a subgroup of 213 patients with the combination of a primary diagnosis of AIS and secondary diagnosis of Covid-19 compared to 68,700 non-infected AIS cases during the same time period. The mean age of AIS patients with Covid-19 was non-significantly higher as compared to non-infected AIS patients (76.1 ± 20.0 y vs. 74.0 ± 19.0 y; *p* = 0.107), and there was no difference in gender distribution (F/M: 46.7%/53.3% vs. 47.2%/52.8%; *p* = 0.833).

In-hospital mortality rate was significantly higher in patients with AIS and concurrent Covid-19 compared to non-infected AIS patients (22.5% vs. 7.8%, *p* < 0.001; Fig. 1). Those patients, who were treated on an intensive care unit, had the highest in-hospital mortality rate (42.9%) compared to patients treated on a stroke unit (15.8%; *p* = 0.001) or on a regular ward (25.7%; *p* = 0.064). Furthermore, we identified 172 patients admitted with Covid-19 and concurrent coding of AIS as secondary diagnosis, implicating that the AIS occurred during hospitalization due to Covid-19. In-hospital mortality rate in this subgroup was 49.4% (85/172). Thus, total in-hospital mortality of patients with concurrent Covid-19 and AIS (primary or secondary diagnosis) was 34.5%. Yaghi et al. [3] reported a mortality rate of 63.6% in AIS patients with concurrent Covid-19 in New York, which is substantially higher compared to the mortality rate of those patients in our study. The difference between nationwide data and data from selected tertiary care hospitals might explain the discrepancy in the observed mortality rates.

**Fig. 1:**
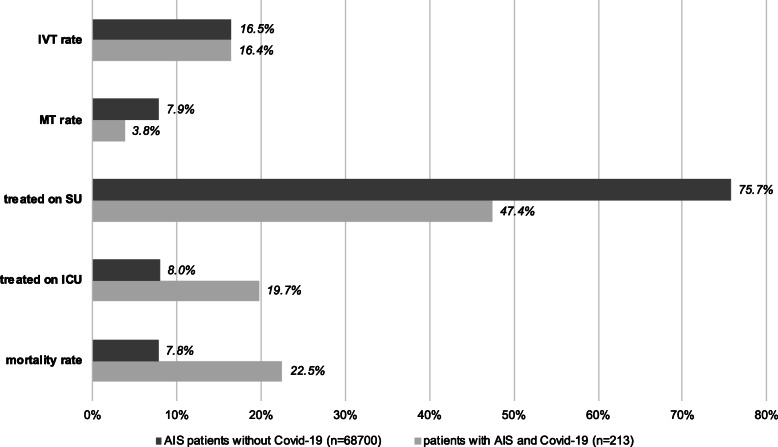
Clinical Characteristics of AIS patients with and without Covid-19. Comparison of treatment rates and in-hospital mortality given in % of patients with primary diagnosis of AIS with and without concurrent Covid-19. Abbreviations: SU = stroke unit; ICU = intensive care unit; IVT = intravenous thrombolysis, MT = mechanical thrombectomy, AIS = acute ischemic stroke. *p < 0.05 (χ2)

In patients with primary diagnosis of AIS and concurrent Covid-19, the intravenous thrombolysis (IVT) rate was 16.4% and the mechanical thrombectomy (MT) rate was 3.8%. Compared to the treatment rates of AIS patients without Covid-19, the MT rate was significantly lower (3.8% vs. 7.9%, *p* = 0.017), while the IVT rate did not differ (16.4 vs. 16.5%, *p* = 0.985; Fig. 1). The study of Yaghi et al. [3] revealed a high rate (45.5%) of large vessel occlusions (LVO) in AIS patients with Covid-19 and a MT rate of 18.8%. Although we are not able to determine the rate of LVO in this nationwide cohort due to missing data from imaging studies, we found a much lower rate for MT in these German AIS patients with concurrent Covid-19 which was also significantly lower as compared to the MT rate in non-infected AIS patients (Fig. 1). Although speculative, since the IVT rates were comparable between stroke patients with and without a concurrent diagnosis of Covid-19, it is unlikely that this finding can be explained by time-depending treatment aspects [4]. It should be noted as a limitation of our analysis, that these administrative data do not provide any information about the severity of symptoms in either AIS or Covid-19. Also, there is no information about testing and allocation strategies in the hospitals which might have varied locally and over time.

Cerebrovascular events have been described to occur more frequently in patients with Covid-19 compared to other viral infections such as influenza [1]. We found a total rate of 1.2% for AIS among hospitalized Covid-19 patients, which is slightly lower compared to a recent meta-analysis performed by Tsivgoulis et al. [5] reporting a stroke rate of 1.6% in Covid-19 patients. However, the previously published prevalences are usually based on registry data from some tertiary care hospitals, which usually also serve as reference hospitals for Covid-19 admissions. Again, this might explain the difference to our nationwide analysis which covers data from a total of 1463 hospitals in Germany.

In summary, these nationwide data point out differences in mortality and medical treatment regime between AIS patients with and without concurrent Covid-19. Since the pandemic is still ongoing, these data draw attention to AIS as a less frequent but often fatal comorbidity in Covid-19 patients.

## Data Availability

Data transmission according to §21 KHEntgG and §24 para. 2 KHG; official data on file, source:, InEK, www.g-drg.de.

## Abbreviations

Covid-19: Coronavirus disease 2019
AIS: Acute ischemic stroke
ICD: International Statistical Classification of Diseases and Related Health Problems
IVT: Intravenous thrombolysis
MT: Mechanical thrombectomy
LVO: Large vessel occlusion
